# Dataset of concentrations of free terpenes at different phenological stages in *Vitis vinifera* L. Shiraz, Cabernet Sauvignon, Riesling, Chardonnay and Pinot Gris

**DOI:** 10.1016/j.dib.2019.104595

**Published:** 2019-10-03

**Authors:** Jiaqiang Luo, Jessica Brotchie, Meng Pang, Philip John Marriott, Kate Howell, Pangzhen Zhang

**Affiliations:** a School of Agriculture and Food, Faculty of Veterinary & Agricultural Sciences, The University of Melbourne, Royal Parade, Parkville, Victoria, 3010, Australia; b School of Chemistry, Monash University Clayton, Victoria, 3800, Australia

**Keywords:** Shiraz, Cabernet Sauvignon, Riesling, Chardonnay, Pinot Gris, terpene, Evolution, SPME-GC-MS

## Abstract

Five *Vitis vinifera* L. cultivars Shiraz, Cabernet Sauvignon, Riesling, Chardonnay and Pinot Gris at different E-L development stages were harvested in two experimental vintages. Temperature and rainfall data of the growing period were obtained from the Australian Government Bureau of Meteorology. Free terpene concentrations of all harvested grape samples were analysed using HS-SPME-GC-MS. One-way ANNOVA was performed to evaluate the significance of changes in terpene concentrations at different maturation stages. More analysis of the data is provided in “Free terpene evolution during the berry maturation of five *Vitis vinifera* L. cultivars” [1].

**Table:** Specifications Table

Subject area	*Phytochemistry, Plant Science*
More specific subject area	Aroma chemistry of wine grapes
Type of data	Figure, Table
How data were acquired	Australian Government Bureau of Meteorology and Gas chromatography-mass spectrometry, experimental results
Data format	Raw data (temperature and rainfall) and analysed data (GC-MS)
Experimental factors	Powdered grape samples were extracted with the extraction solution and the solution sampled by headspace solid-phase microextraction.
Experimental features	Samples were collected at fortnight intervals from four weeks post-flowering (wpf) until commercial harvest. HS-SPME-GC-MS was performed to investigate free terpene concentrations of samples of five varieties with different maturity.
Data source location	Mount Langi Ghiran vineyard, Bayindeen, Victoria, Australia (S 37.316071, E143.145032)
Data accessibility	Data is with this article and a supplemental file containing two data files
Related research article	J. Luo, J. Brotchie, M. Pang, PJ. Marriott, K. Howell, P. Zhang, Free terpene evolution during the berry maturation of five Vitis Vinifera L. cultivars, Food Chem. 299 (2019), 125101

**Table:** 

**Value of the data** • Our previous data [2] showed changes in terpene accumulation during ripening of Shiraz wine grapes. The datasets here provide information about free terpene concentrations at different development stages of Vitis vinifera L. cv. Shiraz, Cabernet Sauvignon, Riesling, Chardonnay and Pinot Gris. • All grape cultivars are located within the same vineyard, which minimizes site variations of environmental and geography factors, which may alter terpene production amongst different cultivars. • These datasets could provide new insights into the free terpene evolution of five economically important wine grape varieties. Further studies could be conducted to investigate the genetic or metabolic differences among cultivars leading to the variations in terpene production.

## 1 Data

**Fig. 1:**
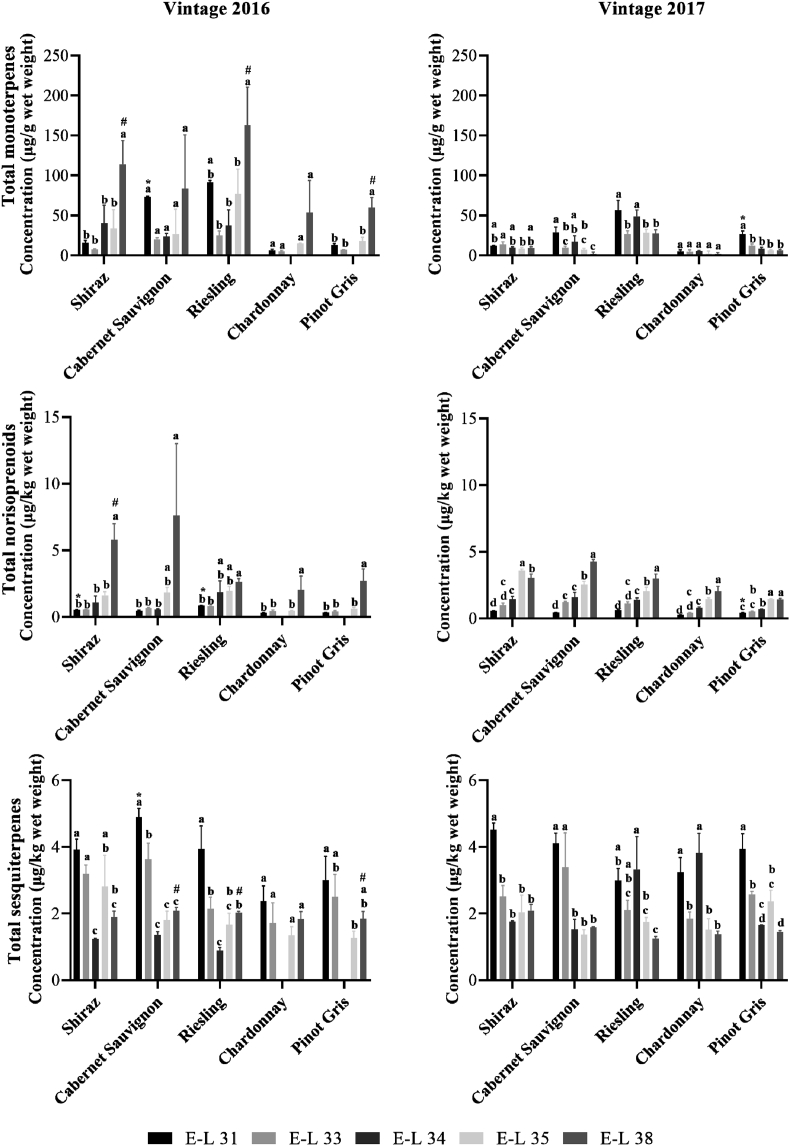
Total terpene contents of five varieties of wine grapes in two vintages. (a) total monoterpene contents in vintage 2016, (b) total monoterpene contents in vintage 2017, (c) total norisoprenoid contents in vintage 2016, (d) total norisoprenoid contents in vintage 2017, (e) total sesquiterpene contents in vintage 2016, (f) total sesquiterpene contents in vintage 2017. Values labelled with the same lower case letter are not significantly (p < 0.05) different within each variety in a vintage. Harvest concentrations of each variety in two vintages labelled with “#”are significantly (p < 0.05) different. Concentrations at E-L 31 of each variety in two vintages labelled with “*” are significantly (p < 0.05) different. Raw data of the histograms are provided in data file 1.

This present data provide supplementary information to our previous work [1]. Total monoterpene, norisoprenoid and sesquiterpene concentrations at E-L 31, 33, 34, 35 and 38 of each variety in two vintages are plotted in Fig. 1. Temperature and rainfall information of vintages 2016 and 2017 was obtained from the Australian Government Bureau of Meteorology (nearest weather station: Ararat Prison Station, BoM ID: 089085, 15.5 km northwest to the experimental vineyard) and summarized in Table 1. Information of growing degree days (GDD) of each sample collection day is provided in Table 2. Compound identification based on comparison of retention indices and mass spectra are summarized in Table 3 and Table 4, respectively. Concentrations of different classes of free terpenes and total monoterpene and total sesquiterpene in different grape cultivars at different developmental stages are shown in Table 5, Table 6, Table 7, Table 8, Table 9.

**Table 1:** Temperature and rainfall conditions during vintages 2016 and 2017.^a^

	January	February	March	April
Vintage 2016				
Mean maximum temperature (^o^C)	30	28.6	27.4	21.9
Mean minimum temperature (^o^C)	11.1	13	12.3	8.3
Mean rainfall (mm)	30	50.6	32	33.4
Solar radiation (MJ m^−2^)	24.1	23.0	16.7	12.8
Vintage 2017				
Mean maximum temperature (^o^C)	29	27.2	27.7	20.7
Mean minimum temperature (^o^C)	12.4	10.8	12	8.4
Mean rainfall (mm)	33	18.6	20	70
Solar radiation (MJ m^−2^)	24.4	21.5	17.3	10.9

a Data were obtained from the website of the Australian Government Bureau of Meteorology using the Ararat Prison observation station data.

**Table 2:** Accumulated growing degree days (GDD)* of sample collection dates. Raw data of the table are provided in data file 2.

Vintage 2016		Vintage 2017	
Date	GDD	Date	GDD
18/12/2015	583.35	9/01/2017	584.25
2/01/2016	757	23/01/2017	726.05
15/01/2019	898.1	10/02/2017	913.8
1/02/2016	1078.1	20/02/2017	983.85
13/02/2016	1208.05	6/03/2017	1128.75
29/02/2016	1356.75	20/03/2017	1273.3
14/03/2016	1531.95	3/04/2007	1369.95
		18/04/2017	1438.05

*GDD of each sample collection date is calculated based on the following equation [5]:$\text{GDD}=\sum_{1\;Oct}^{date\;of\;sample\;collection}\text{max}\left[\left(\left(\frac{T\text{max}+T\text{min}}{2}\right)-10\right);0\right]$

Where,

*T*max is the highest temperature of the sample collection day.

*T*min is the lowest temperature of the sample collection day.

**Table 3:** Retention indices relative to *n*-alkanes C7–C30 on a J&W DΒ-5ms column, reference retention indices, and target ions of identified terpenoids.

	Calculated RI (DΒ-5)	Terpenoids library RI (DΒ-1)^a^	NIST library RI (DΒ-5)	Target ions^b^
*Monoterpenoids*				
α-Terpinene	1013	1013	1018	**121**,93,136
Cymene (m- and p-)	1022	1013	1026	**119**,91,134
1,8-Cineol	1130	1024	1031	**81**,108,71,154
(E)-β-Ocimene	1044	1041	1026	**93**,121,79
γ-Terpinene	1055	1051	1060	**93**,136,121
Terpinolene	1082	1082	1088	**136**,93,121
p-Cymenene	1086	1075	1080	**132**,117,91
Linalool	1097	1086	1098	**71**,93,55,121
Hotrienol	1101	1083	1104	**71**,82,67
trans-Pinocarveol	1125	1126	1137	**55**,70,83
Citronellal	1129	1129	1150	**69**,55,111
Neroloxide	1147	1137	1153	**68**,83,96
Menthol (+isomenthol)	1172&1175	1172&1176	1174	**71**,81,95
Terpinen-4-ol	1175	1164	1179	**71**,93,111,154
α-Terpineol	1192	1176	1189	**59**,93,121,136
Geraniol	1246	1235	1255	**69**,41,93,53
Geranylacetone	1442	1430	1452	**69**,107,93,151
*Norisoprenoids*				
Theaspirane (Isomer 1)	1292	1299	1298	**138**,96,82
Theaspirane (Isomer 2)	1310	1313	1298	**138**,109,82
(*E*)-β-Damascenone	1372	1363	1385	**121**,190,69
*Sesquiterpenoids*				
7-epi-α-Cedrene	1407	1404	1405	**119**,93,204
Selina-4,11-diene	1467	1475	NA	**189**,204,81
α-Muurolene	1491	1491	1499	**105**,161,119,204
δ-Cadinene	1510	1520	1510	**161**,119,204
Calamenene (cis + trans)	1513	1517	1521	**159**,202,114
α-Calacorene	1526	1527	1523	**157**,142,200
ω-Cadinene	1526	1526	1528	**119**,161,204
γ-Calacorene	1533	1554	1550	**157**,142,200
Palustrol	1565	1569	1568	**111**,204,161
1-epi-Cubenol	1620	1623	1625	**119**,161,204
γ-Eudesmol	1624	1618	1630	**189**,204,161
Cubenol	1633	1630	1643	**161**,105,69

a Only RI values from a DB-1 column are available in the terpenoids library. RI profiles of DB-1 and DB-5 are close as demonstrated in previous data [4].

b The first of the target ions was used as quantifier and others were qualifiers.

**Table 4:** Comparisons between reference terpenoid mass spectra from the terpenoids library (upper frame) and for an experimental peak in a representative sample (lower frame).

Terpenoid	Mass spectrum
*Monoterpenoids*	
α-Terpinene	
Cymene (m- & p-)	
1,8-Cineol	
(*E*)-β-Ocimene	
γ-Terpinene	
Terpinolene	
p-Cymenene	
Linalool	
Hotrienol	
trans-Pinocarveol	
Citronellal	
Neroloxide	
Menthol (+isomenthol)	
α-Terpineol	
γ-Terpineol	
Geraniol	
Geranylacetone	
*Norisoprenoids*	
Theaspirane (Isomer 1)	
Theaspirane (Isomer 2)	
(*E*)-β-Damascenone	
Sesquiterpenoids	
7-epi-α-Cedrene	
Selina-4,11-diene	
α-Muurolene	
δ-Cadinene	
Calamenene (cis + trans)	
α-Calacorene	
ω-Cadinene	
γ-Calacorene	
Palustrol	
1-epi-Cubenol	
γ-Eudesmol	
Cubenol	
Cadalene

**Table 5:** Terpene concentrations at different developmental stages of Shiraz in the two experimental vintages.

Vintage 2016	wpf4	wpf6	wpf8	wpf10	wpf12	wpf14	wpf16
Monterpenoids							
α-Terpinene	<0.01	<0.01	<0.01	0.31 ± 0.08	<0.01	<0.01	<0.01
Cymene (m- and p-)	<0.01	<0.01	<0.01	0.62 ± 0.31a	0.65 ± 0.19a	0.5 ± 0.15a	1.17 ± 0.56a
1,8-Cineol	ND	ND	ND	ND	ND	ND	ND
(*E*)-β-Ocimene	<0.01	ND	ND	ND	ND	ND	ND
γ-Terpinene	ND	ND	ND	ND	ND	ND	ND
Terpinolene	<0.01	<0.01	<0.01	0.25 ± 0.13	<0.01	<0.01	<0.01
p-Cymenene	<0.01	<0.01	<0.01	<0.01	<0.01	<0.01	0.22 ± 0.16
Linalool	2.25 ± 0.2bc	0.78 ± 0.16c	2.59 ± 2.73abc	0.8 ± 0.56c	10.68 ± 3.42ab	6.63 ± 4.32abc	12.06 ± 6.9a
Hotrienol	<0.01	<0.01	0.89 ± 0.94b	0.11 ± 0.06b	4.19 ± 0.87a	1.41 ± 0.12b	0.49 ± 0.41b
trans-Pinocarveol	<0.01	<0.01	0.94 ± 0.46c	7.17 ± 0.79bc	11.7 ± 1.13ab	9.06 ± 5.92bc	20.08±2a
Citronellal	0.17 ± 0.08c	<0.01	1.5 ± 0.56c	12.27 ± 1.42bc	19.05 ± 1.72ab	15.56 ± 9.55b	31.52 ± 3.72a
Neroloxide	<0.01	<0.01	<0.01	<0.01	0.46 ± 0.29	<0.01	<0.01
Menthol (+isomenthol)	<0.01	<0.01	<0.01	3.57 ± 0.5b	5.35 ± 0.54ab	3.78 ± 2.88b	9.09 ± 1.03a
Terpinen-4-ol	<0.01	<0.01	<0.01	0.07 ± 0.06b	<0.01	<0.01	0.38 ± 0.27a
α-Terpineol	2.2 ± 0.06bc	0.48 ± 0.1c	1.87 ± 1.2bc	2.35 ± 0.02bc	4.38 ± 0.52ab	3.22 ± 2.38abc	6.43 ± 1.32a
Geraniol	1.44 ± 0.26bc	1.44 ± 0.07bc	2.62 ± 1.52abc	1.06 ± 0.17c	6.17 ± 1.59a	5.25 ± 2.26a	4.99 ± 1.3ab
Geranylacetone	9.35 ± 2.98a	4.66 ± 0.86a	31.31 ± 14.36a	14.2 ± 7.37a	24.02 ± 6.86a	21.46 ± 16.1a	27.49 ± 14.32a
Total	15.48 ± 3.45c	7.46 ± 0.88c	40.15 ± 22.56bc	33.92 ± 23.52bc	86.63 ± 10.44ab	66.23 ± 44.01abc	113.8 ± 30.13a
*Norisoprenoids*							
Theaspirane (Isomer 1)	0.18 ± 0.01ab	0.12 ± 0.01ab	0.25 ± 0.16ab	0.05±0b	0.36 ± 0.06a	0.15 ± 0.09ab	0.2 ± 0.18ab
Theaspirane (Isomer 2)	0.12±0ab	0.1 ± 0.01ab	0.18 ± 0.1a	0.04±0b	0.18 ± 0.02a	0.08 ± 0.05ab	0.09 ± 0.05ab
(*E*)-β-Damascenone	0.24±0c	0.34 ± 0.03c	0.65 ± 0.26c	1.53 ± 0.28bc	4.59 ± 0.35a	3.84 ± 1.95ab	5.5 ± 0.97a
Total	0.54 ± 0.01c	0.56 ± 0.03c	1.08 ± 0.51c	1.61 ± 0.28bc	5.13 ± 0.39a	4.08 ± 2.08ab	5.79 ± 1.2a
*Sesquiterpenoids*							
7-epi-α-Cedrene	1.71 ± 0.3ab	2.24 ± 0.23a	0.72 ± 0.02d	1.57 ± 0.48bc	0.96 ± 0.02cd	1 ± 0.03cd	1 ± 0.06cd
Selina-4,11-diene	0.12 ± 0.01a	0.05±0b	0.03 ± 0.01c	0.02±0c	ND	ND	ND
α-Muurolene	0.13 ± 0.01a	0.14±0a	0.05 ± 0.01b	0.06 ± 0.03b	ND	ND	ND
δ-Cadinene	ND	ND	ND	0.12 ± 0.1a	0.11 ± 0.03a	0.17 ± 0.07a	0.18 ± 0.04a
Calamenene (cis + trans)	0.29 ± 0.01a	0.12 ± 0.01b	0.07 ± 0.01b	0.15 ± 0.07b	0.1 ± 0.03b	0.07 ± 0.02b	0.1 ± 0.02b
α-Calacorene	0.08 ± 0.02a	0.03±0ab	0.02 ± 0.01b	0.06 ± 0.04ab	0.04 ± 0.02ab	0.03 ± 0.02ab	0.05 ± 0.02ab
ω-Cadinene	0.21 ± 0.02a	0.08 ± 0.01b	0.04 ± 0.01b	0.07 ± 0.02b	0.04 ± 0.02b	0.06 ± 0.02b	0.07 ± 0.02b
γ-Calacorene	0.76 ± 0.01a	0.33 ± 0.03bc	0.13 ± 0.05d	0.37 ± 0.14b	0.15 ± 0.03cd	0.16 ± 0.07cd	0.21 ± 0.04bcd
Palustrol	0.13 ± 0.02a	0.01±0b	0.02 ± 0.01b	0.03 ± 0.02b	0.03 ± 0.01b	0.06 ± 0.03b	ND
1-epi-Cubenol	0.16 ± 0.01a	0.03 ± 0b	0.04 ± 0.01b	0.09 ± 0.06ab	0.1 ± 0ab	0.1 ± 0.02ab	0.14 ± 0.07a
γ-Eudesmol	0.13 ± 0.01a	0.03 ± 0b	0.02 ± 0b	0.1 ± 0.07ab	ND	ND	ND
Cubenol	0.08 ± 0.01a	0.03 ± 0.01a	0.03 ± 0.01a	0.07 ± 0.05a	0.05 ± 0.02a	0.07 ± 0.02a	0.09 ± 0.06a
Cadalene	0.13 ± 0.01a	0.11 ± 0.01a	0.05 ± 0.02c	0.09 ± 0.02ab	0.03 ± 0.01c	0.04 ± 0.02c	0.05 ± 0.02bc
Total	3.92 ± 0.31a	3.19 ± 0.27a	1.23 ± 0.03c	2.81 ± 0.94ab	1.61 ± 0.09c	1.78 ± 0.14bc	1.89 ± 0.18bc

Vintage 2017								
*Monterpenoids*								
α-Terpinene	<0.01	<0.01	<0.01	<0.01	<0.01	<0.01	<0.01	<0.01
Cymene (m- and p-)	<0.01	<0.01	<0.01	<0.01	<0.01	<0.01	<0.01	<0.01
1,8-Cineol	ND	ND	ND	ND	ND	ND	ND	ND
(*E*)-β-Ocimene	0.14 ± 0.09	<0.01	ND	ND	ND	<0.01	<0.01	<0.01
γ-Terpinene	ND	ND	ND	ND	ND	ND	ND	ND
Terpinolene	<0.01	<0.01	<0.01	<0.01	<0.01	<0.01	<0.01	<0.01
p-Cymenene	<0.01	<0.01	<0.01	<0.01	<0.01	<0.01	<0.01	<0.01
Linalool	1.03 ± 0.31bcd	1.6 ± 0.56ab	0.57 ± 0.12cd	0.39 ± 0.13d	0.94 ± 0.13bcd	1.14 ± 0.16bc	1.18 ± 0.21abc	1.89 ± 0.11a
Hotrienol	1.11 ± 0.87b	5.63 ± 1.64a	5.46 ± 1.45a	3.37 ± 1.76ab	2.47 ± 0.74ab	0.99 ± 0.24b	0.19 ± 0.02b	0.27 ± 0.18b
trans-Pinocarveol	<0.01	<0.01	<0.01	<0.01	<0.01	<0.01	<0.01	<0.01
Citronellal	<0.01	<0.01	<0.01	<0.01	<0.01	0.22 ± 0.1a	0.08 ± 0.04b	0.26 ± 0.14a
Neroloxide	<0.01	<0.01	<0.01	<0.01	<0.01	<0.01	<0.01	<0.01
Menthol (+isomenthol)	<0.01	<0.01	<0.01	<0.01	<0.01	<0.01	<0.01	<0.01
Terpinen-4-ol	<0.01	<0.01	<0.01	<0.01	<0.01	<0.01	<0.01	<0.01
α-Terpineol	<0.01	0.44 ± 0.2ab	0.23 ± 0.02b	<0.01	0.37 ± 0.17ab	0.53 ± 0.33ab	0.57 ± 0.11ab	0.77 ± 0.13a
Geraniol	1.36 ± 0.18d	1.18 ± 0.29d	0.9 ± 0.2d	1.14 ± 0.04d	1.39 ± 0.18d	1.96 ± 0.19c	3.72 ± 0.19a	2.62 ± 0.14b
Geranylacetone	8.54 ± 0.75a	8.54 ± 0.22a	6.64 ± 2.76ab	4.85 ± 1.04ab	2.98 ± 1.73b	3.17 ± 1.78b	5.18 ± 2.44ab	3.32 ± 1.67b
Total	12.21 ± 0.43abc	17.62 ± 2.82a	13.98 ± 3.16ab	9.76 ± 1.25bc	8.15 ± 1.78bc	7.94 ± 2.19c	10.86 ± 2.15bc	9.05 ± 1.92bc
*Norisoprenoids*								
Theaspirane (Isomer 1)	0.15 ± 0a	0.16 ± 0.03a	0.11 ± 0.02b	0.08 ± 0.01bc	0.06 ± 0cd	0.05 ± 0cd	0.04 ± 0d	0.04 ± 0d
Theaspirane (Isomer 2)	0.14 ± 0.01a	0.13 ± 0.02a	0.08 ± 0.01b	0.07±0bc	0.05 ± 0cd	0.04 ± 0d	0.03 ± 0d	0.03 ± 0d
(*E*)-β-Damascenone	0.3 ± 0.02f	0.49 ± 0.11ef	0.82 ± 0.13de	1.29 ± 0.21d	3.49 ± 0.1a	2.77 ± 0.05b	1.95 ± 0.24c	2.97 ± 0.29b
Total	0.58 ± 0.01e	0.78 ± 0.16e	1.01 ± 0.15de	1.44 ± 0.22d	3.6 ± 0.1a	2.85 ± 0.05b	2.02 ± 0.24c	3.03 ± 0.3b
*Sesquiterpenoids*								
7-epi-α-Cedrene	3.18 ± 0.25a	3.63±1a	1.52 ± 0.25b	1.11 ± 0.06b	1.45 ± 0.47b	1.3 ± 0.15b	1.1 ± 0.12b	1.11 ± 0.19b
Selina-4,11-diene	0.09 ± 0.01a	0.08 ± 0.03ab	0.05 ± 0.01bc	0.03±0cd	0.03 ± 0.01cd	0.02 ± 0.01cd	0.01±0cd	0.01±0d
α-Muurolene	0.05 ± 0.01bc	0.16 ± 0.04a	0.16 ± 0.03a	0.09 ± 0.02bc	0.1 ± 0.01b	0.06 ± 0.01bc	0.04 ± 0.01c	0.04 ± 0.01c
δ-Cadinene	ND	ND	ND	ND	ND	ND	ND	ND
Calamenene (cis + trans)	0.15 ± 0.02ab	0.2 ± 0.05a	0.15 ± 0.01ab	0.08 ± 0.02c	0.08 ± 0.02c	0.06 ± 0.03c	0.06 ± 0.01c	0.1 ± 0.01bc
α-Calacorene	0.06 ± 0.01a	0.05 ± 0.01ab	0.03 ± 0bc	0.02 ± 0c	ND	ND	ND	ND
ω-Cadinene	0.18 ± 0.02a	0.12 ± 0.02b	0.04±0c	0.04 ± 0.01c	0.03±0c	0.03±0c	0.03±0c	0.03 ± 0.01c
γ-Calacorene	0.42 ± 0.03abc	0.56 ± 0.14a	0.34 ± 0.03bcd	0.22 ± 0.01de	0.2 ± 0.02de	0.18 ± 0.01e	0.28 ± 0.03cde	0.48 ± 0.05ab
Palustrol	0.14 ± 0.03a	0.04± 0b	0.01 ± 0b	0.01 ± 0b	0.01 ± 0b	ND	ND	ND
1-epi-Cubenol	0.09 ± 0.02a	0.06 ± 0.02b	0.04 ± 0bc	0.02 ± 0c	0.02 ± 0c	0.03 ± 0c	0.02 ± 0c	0.02 ± 0.01c
γ-Eudesmol	0.02±0bc	0.04 ± 0.01a	0.03 ± 0ab	0.02 ± 0bc	0.02 ± 0bc	0.02 ± 0bc	0.01 ± 0c	0.02 ± 0.01bc
Cubenol	0.07 ± 0.01a	0.05 ± 0.02a	0.03 ± 0b	0.02 ± 0b	0.02 ± 0.01b	0.03±0b	0.02 ± 0.01b	0.02 ± 0b
Cadalene	0.06±0e	0.17 ± 0.03b	0.12 ± 0.01c	0.08 ± 0.01cde	0.08 ± 0.01cde	0.08±0de	0.1 ± 0.01cd	0.23 ± 0.02a
Total	4.51 ± 0.2a	5.15 ± 1.35a	2.51 ± 0.34b	1.75 ± 0.03b	2.04 ± 0.52b	1.81 ± 0.13b	1.68 ± 0.11b	2.08 ± 0.19b

**Notes:** Linalool, α-terpineol, geraniol and geranylacetone were quantified using their pure standard compounds. α-terpinene, cymene (m- and p-), 1,8-cineol, (*E*)-β-ocimene, γ-terpinene, terpinolene, p-cymenene and hotrienol were semi-quantified using a linalool standard. All monoterpenes are expressed at μg/g grape sample. All sesquiterpenoids and norisoprenoids were semi-quantified with the internal standard β-cedrene and expressed as equivalent concentrations of the internal standard at μg/kg grape sample. ND: not detected. Values labelled with the same lower case letter in the same row are not significantly (*p* < 0.05) different. Raw data of the table are provide in data file 1.

**Table 6:** Terpene concentrations at different developmental stages of Cabernet Sauvignon in the two experimental vintages.

Vintage 2016	wpf4	wpf6	wpf8	wpf10	wpf12	wpf14	wpf16
Monterpenoids							
α-Terpinene	9.17 ± 0.13a	1.02 ± 0.19b	0.2 ± 0.09b	<0.01	0.45 ± 0.2b	0.44 ± 0.04b	1.28 ± 1.67b
Cymene (m- and p-)	2.94 ± 0.03a	0.42 ± 0.12a	<0.01	<0.01	1.04 ± 0.3a	0.95 ± 0.14a	1.8 ± 2.52a
1,8-Cineol	9.12 ± 0.77a	2.28 ± 0.24b	0.55 ± 0.15c	<0.01	ND	ND	ND
(*E*)-β-Ocimene	ND	ND	ND	ND	ND	ND	ND
γ-Terpinene	20.99 ± 0.68a	5.14 ± 0.37b	ND	ND	ND	ND	ND
Terpinolene	5.64 ± 0.09a	0.87 ± 0.01b	0.1 ± 0.05c	<0.01	0.29 ± 0.2c	<0.01	<0.01
p-Cymenene	0.13 ± 0.05a	<0.01	<0.01	<0.01	0.12 ± 0.06a	<0.01	<0.01
Linalool	0.55 ± 0.16a	<0.01	<0.01	0.83 ± 1.01a	4 ± 0.77a	1.73 ± 1.36a	4.72 ± 4.83a
Hotrienol	<0.01	<0.01	<0.01	0.3 ± 0.18a	0.32 ± 0.36a	<0.01	<0.01
trans-Pinocarveol	0.2 ± 0.1b	<0.01	<0.01	<0.01	15.83±3a	6.25 ± 3.73ab	14.3 ± 8.1a
Citronellal	0.37 ± 0.16b	<0.01	<0.01	<0.01	26.19 ± 4.58a	10.6 ± 5.39ab	24.63 ± 12.89a
Neroloxide	<0.01	<0.01	<0.01	<0.01	ND	ND	ND
Menthol (+isomenthol)	<0.01	<0.01	<0.01	<0.01	10.64 ± 1.99a	3.87 ± 2.07a	9.51 ± 6.13a
Terpinen-4-ol	5.66 ± 0.88a	0.18 ± 0.09b	<0.01	<0.01	0.36 ± 0.24b	<0.01	<0.01
α-Terpineol	6.98 ± 0.87a	1.77 ± 0.19ab	0.69 ± 0.23b	<0.01	4.91 ± 0.77ab	3.04 ± 0.09ab	4.58 ± 4.56ab
Geraniol	1.04 ± 0.14a	0.98 ± 0.03a	0.91 ± 0.15a	1.07 ± 0.19a	1.63 ± 0.13a	1.63 ± 0.72a	1.43 ± 0.36a
Geranylacetone	10.46 ± 1.67a	7.42 ± 2.03a	20.88 ± 4.22a	10.79 ± 6.77a	27.24 ± 6.74a	19.31 ± 15.08a	19.54 ± 23.15a
Total	73.19±1a	20.09 ± 2.08a	23.37 ± 4.33a	26.46 ± 31.28a	92.98 ± 17.43a	46.51 ± 30.96a	83.36 ± 67.39a
*Norisoprenoids*							
Theaspirane (Isomer 1)	0.05 ± 0a	0.05 ± 0a	0.04 ± 0.01a	0.07 ± 0.01a	0.06 ± 0.02a	0.05 ± 0.03a	ND
Theaspirane (Isomer 2)	0.04 ± 0.01a	0.05 ± 0.01a	0.04 ± 0.01a	0.05 ± 0a	0.06 ± 0.01a	ND	ND
(*E*)-β-Damascenone	0.37 ± 0.03b	0.55 ± 0.06b	0.47 ± 0.04b	1.72 ± 0.41ab	5.84 ± 0.82ab	4.95 ± 2.44ab	7.6 ± 5.42a
Total	0.47 ± 0.04b	0.65 ± 0.05b	0.56 ± 0.06b	1.83 ± 0.39ab	5.96 ± 0.83ab	5 ± 2.46ab	7.6 ± 5.42a
*Sesquiterpenoids*							
7-epi-α-Cedrene	2.13 ± 0.03a	2.53 ± 0.46a	0.82 ± 0.05b	1.13 ± 0.19b	0.94 ± 0.03b	1.05 ± 0.1b	1.08 ± 0.06b
Selina-4,11-diene	0.08 ± 0.01a	0.02 ± 0b	0.02 ± 0b	0.02 ± 0.01b	ND	ND	ND
α-Muurolene	0.07 ± 0.01a	0.07 ± 0.01a	0.04 ± 0.01a	0.07 ± 0.04a	ND	ND	ND
δ-Cadinene	ND	ND	ND	ND	0.12 ± 0.01a	0.15 ± 0.05a	0.19 ± 0.03a
Calamenene (cis + trans)	0.46 ± 0.04a	0.17 ± 0.01b	0.08±0c	0.09 ± 0.01c	0.1 ± 0.02bc	0.07 ± 0.01c	0.1 ± 0.05bc
α-Calacorene	0.12 ± 0.03a	0.04 ± 0.01b	0.02±0b	0.03 ± 0.02b	0.04±0b	0.04 ± 0.01b	0.06 ± 0.02b
ω-Cadinene	0.24 ± 0.01a	0.08 ± 0.01b	0.03±0c	0.05 ± 0.01bc	0.07 ± 0.01bc	0.05 ± 0.01bc	0.06 ± 0.04bc
γ-Calacorene	1.18 ± 0.1a	0.45 ± 0.04b	0.23 ± 0.06c	0.21 ± 0.04c	0.22 ± 0.01c	0.21 ± 0.06c	0.24 ± 0.08c
Palustrol	0.2 ± 0.03a	0.02 ± 0b	ND	ND	0.07 ± 0.01b	0.05 ± 0.02b	0.06±0b
1-epi-Cubenol	0.15 ± 0.01ab	0.04 ± 0.01bc	0.03 ± 0d	0.06 ± 0.07bcd	0.17 ± 0.03a	0.12 ± 0.05abc	0.15 ± 0.02ab
γ-Eudesmol	0.07 ± 0.01a	0.03 ± 0b	0.01 ± 0c	0.02 ± 0.01bc	ND	ND	ND
Cubenol	0.07 ± 0.01abc	0.03 ± 0bc	0.02 ± 0c	0.04 ± 0.04abc	0.11 ± 0.02a	0.09 ± 0.03ab	0.08 ± 0.03abc
Cadalene	0.12 ± 0a	0.13 ± 0.02a	0.06 ± 0b	0.08 ± 0.01b	0.05 ± 0b	0.05 ± 0.01b	0.06 ± 0.02b
Total	4.89 ± 0.26a	3.62 ± 0.49b	1.36 ± 0.1d	1.8 ± 0.27cd	1.87 ± 0.09cd	1.89 ± 0.14cd	2.07 ± 0.11c

**Vintage 2017**	2	7	12	17	22	27	32	37
	wpf4	wpf6	wpf8	wpf10	wpf12	wpf14	wpf16	wpf18
*Monterpenoids*								
α-Terpinene	3.12 ± 0.95a	2.51 ± 0.29a	0.81 ± 0.32b	0.95 ± 0.06b	<0.01	<0.01	<0.01	<0.01
Cymene (m- and p-)	0.85 ± 0.4ab	1.25 ± 0.22a	0.39 ± 0.17b	0.62 ± 0.25ab	<0.01	<0.01	<0.01	<0.01
1,8-Cineol	8.83 ± 2.08a	6.28 ± 1.07a	1.64 ± 0.44b	2.19 ± 1.67b	<0.01	ND	ND	ND
(*E*)-β-Ocimene	<0.01	ND	ND	ND	ND	ND	ND	ND
γ-Terpinene	7.39 ± 1.87a	8.91 ± 1.15a	ND	ND	ND	ND	ND	ND
Terpinolene	1.68 ± 0.38a	1.45 ± 0.25a	0.31 ± 0.12b	0.42 ± 0.16b	<0.01	<0.01	<0.01	<0.01
p-Cymenene	<0.01	<0.01	<0.01	<0.01	<0.01	<0.01	<0.01	<0.01
Linalool	0.45 ± 0.18a	0.25 ± 0.21a	<0.01	0.74 ± 0.66a	0.29 ± 0.17a	0.12 ± 0.1a	0.12 ± 0.09a	0.11 ± 0.03a
Hotrienol	0.21 ± 0.17a	0.81 ± 0.32a	0.22 ± 0.08a	0.91 ± 0.46a	<0.01	<0.01	<0.01	<0.01
trans-Pinocarveol	<0.01	<0.01	<0.01	0.23 ± 0.08	<0.01	<0.01	<0.01	<0.01
Citronellal	<0.01	<0.01	<0.01	0.4 ± 0.47a	0.16 ± 0.1a	0.21 ± 0.17a	0.09 ± 0.03a	0.21 ± 0.07a
Neroloxide	<0.01	<0.01	<0.01	<0.01	<0.01	<0.01	ND	ND
Menthol (+isomenthol)	<0.01	<0.01	<0.01	<0.01	<0.01	<0.01	<0.01	<0.01
Terpinen-4-ol	0.96 ± 0.34a	0.72 ± 0.34a	<0.01	<0.01	<0.01	<0.01	<0.01	<0.01
α-Terpineol	0.73 ± 0.09ab	1.46 ± 0.33a	0.41 ± 0.05b	1.33 ± 0.49a	0.06 ± 0.04b	0.32 ± 0.39b	0.4 ± 0.14b	0.18 ± 0.1b
Geraniol	0.71 ± 0.03d	0.73 ± 0.06d	0.62 ± 0.05d	0.72 ± 0.07d	1.73 ± 0.08a	1.07 ± 0.19c	1.39 ± 0.12b	1.25 ± 0.13bc
Geranylacetone	3.83 ± 1.39ab	5.77 ± 2.07ab	5.57 ± 1.03ab	8.76 ± 4.31a	4.85 ± 1.89ab	1.21 ± 2.12b	0.95 ± 1.68b	0.39 ± 2.05b
Total	28.76 ± 6.78ab	30.23 ± 4.51a	9.91 ± 1.58cd	17.01 ± 7.63bc	7.02 ± 2.02cd	2.88 ± 2.42d	2.79 ± 1.77d	2.07 ± 2.15d
*Norisoprenoids*								
Theaspirane (Isomer 1)	0.03 ± 0.01bc	0.06 ± 0.01a	0.05 ± 0.01ab	0.05 ± 0.02a	0.03±0bc	0.02±0c	0.01 ± 0.01c	0.01 ± 0c
Theaspirane (Isomer 2)	0.03 ± 0ab	0.05 ± 0.02a	0.03 ± 0ab	0.05 ± 0.02a	0.03 ± 0ab	0.02 ± 0.01b	0.02 ± 0.01b	0.01 ± 0b
(*E*)-β-Damascenone	0.37 ± 0.02e	0.91 ± 0.09de	1.13 ± 0.06de	1.47 ± 0.36d	2.46 ± 0.27c	3.51 ± 0.35ab	3.07 ± 0.54bc	4.23 ± 0.18a
Total	0.44 ± 0.02e	1.02 ± 0.11de	1.21 ± 0.07de	1.57 ± 0.39d	2.51 ± 0.27c	3.55 ± 0.36ab	3.1 ± 0.54bc	4.26 ± 0.18a
*Sesquiterpenoids*								
7-epi-α-Cedrene	2.74 ± 0.25ab	3.5 ± 1.77a	2.42 ± 0.87ab	1.12 ± 0.19b	1.06 ± 0.14b	1.57 ± 0.16ab	1.04 ± 0.05b	1.27 ± 0.02b
Selina-4,11-diene	0.05 ± 0.01a	0.04 ± 0.01ab	0.03 ± 0b	ND	ND	ND	ND	ND
α-Muurolene	0.03 ± 0.01b	0.06±0b	0.05 ± 0.01ab	ND	ND	ND	ND	ND
δ-Cadinene	ND	ND	ND	ND	ND	ND	ND	ND
Calamenene (cis + trans)	0.17 ± 0.04ab	0.24 ± 0.03a	0.16 ± 0.04b	0.11 ± 0.01bc	0.05±0c	0.08 ± 0.01c	0.05 ± 0.01c	0.06 ± 0.01c
α-Calacorene	0.07 ± 0.02a	0.07 ± 0.01a	0.03 ± 0.01b	ND	ND	ND	ND	ND
ω-Cadinene	0.19 ± 0.03a	0.11 ± 0.02b	0.05 ± 0.01c	ND	ND	ND	ND	ND
γ-Calacorene	0.5 ± 0.06b	0.78 ± 0.05a	0.46 ± 0.06b	0.18 ± 0.09c	0.19 ± 0.01c	0.24 ± 0.03c	0.18 ± 0.02c	0.2 ± 0.01c
Palustrol	0.16 ± 0.03a	0.04 ± 0.01b	0.01±0c	ND	ND	ND	ND	ND
1-epi-Cubenol	0.07 ± 0.02a	0.06 ± 0.01a	0.02 ± 0.01b	0.02 ± 0b	0.02 ± 0b	0.02 ± 0b	0.01 ± 0b	ND
γ-Eudesmol	0.01 ± 0b	0.02 ± 0a	0.02 ± 0.01a	0.01±0ab	ND	ND	ND	ND
Cubenol	0.06 ± 0.02a	0.04 ± 0.01ab	0.02 ± 0.01b	0.02±0b	ND	ND	ND	ND
Cadalene	0.04 ± 0.01c	0.18 ± 0.02a	0.11 ± 0.02b	0.05 ± 0.03c	0.04 ± 0c	0.07 ± 0c	0.05 ± 0c	0.06 ± 0.01c
Total	4.11 ± 0.31ab	5.14 ± 1.91a	3.39 ± 1.03abc	1.52 ± 0.31c	1.37 ± 0.14c	1.98 ± 0.16bc	1.33 ± 0.07c	1.59 ± 0.01c

**Notes:** Linalool, α-terpineol, geraniol and geranylacetone were quantified using their pure standard compounds. α-terpinene, cymene (m- and p-), 1,8-cineol, (*E*)-β-ocimene, γ-terpinene, terpinolene, p-cymenene and hotrienol were semi-quantified using a linalool standard. All monoterpenes are expressed at μg/g grape sample. All sesquiterpenoids and norisoprenoids were semi-quantified with the internal standard β-cedrene and expressed as equivalent concentrations of the internal standard at μg/kg grape sample. ND: not detected. Values labelled with the same lower case letter in the same row are not significantly (*p* < 0.05) different. Raw data of the table are provide in data file 1.

**Table 7:** Terpene concentrations at different developmental stages of Riesling in the two experimental vintages.

Vintage 2016	wpf4	wpf6	wpf8	wpf10	wpf12	wpf14
*Monterpenoids*						
α-Terpinene	9.87 ± 0.24a	1.82 ± 0.47b	1.43±1b	0.59 ± 0.17b	0.61 ± 0.31b	0.53 ± 0.4b
Cymene (m- and p-)	6.03 ± 0.12a	0.86 ± 0.48b	1.95 ± 1.45b	0.45 ± 0.36b	0.96 ± 0.61b	0.11 ± 0.13b
1,8-Cineol	0.64 ± 0.27	<0.01	<0.01	ND	ND	ND
(*E*)-β-Ocimene	<0.01	<0.01	ND	ND	1.51 ± 0.58b	4.6 ± 2.07a
γ-Terpinene	26.79 ± 1.35a	7.33 ± 0.85b	ND	ND	ND	ND
Terpinolene	10.38 ± 0.1a	2.9 ± 0.71c	2.19 ± 1.18c	0.79 ± 0.23c	2.96 ± 0.64bc	6.31 ± 2.59b
p-Cymenene	0.73 ± 0.08a	<0.01	0.43 ± 0.01b	<0.01	<0.01	<0.01
Linalool	3.8 ± 0.38c	1.17 ± 0.24c	2.87 ± 2.06c	6.03 ± 2.53c	29.19 ± 8.44b	58.55 ± 15.31a
Hotrienol	1.33 ± 0.33c	0.8 ± 0.54c	1.85 ± 1.3c	9.97 ± 5.1bc	20.38 ± 1.81ab	35.5 ± 12.34a
trans-Pinocarveol	0.23 ± 0.02a	0.16±0a	0.56 ± 0.38a	10.07 ± 6.5a	3.42 ± 1.31a	1.91 ± 0.31a
Citronellal	0.59 ± 0.51b	0.73 ± 0.06b	1.41 ± 0.56b	16.11 ± 8.58a	6.15 ± 1.56ab	4.17 ± 0.31ab
Neroloxide	<0.01	<0.01	<0.01	1.12 ± 0.74b	5.61 ± 1.16ab	7.87 ± 3.25a
Menthol (+isomenthol)	<0.01	<0.01	0.09 ± 0.07b	5.89 ± 4.11a	2.01 ± 0.8ab	1.12 ± 0.21ab
Terpinen-4-ol	6.99 ± 0.03a	0.82 ± 0.29b	1.18 ± 0.5b	<0.01	<0.01	<0.01
α-Terpineol	17.14 ± 0.3ab	5.43 ± 0.82c	5.96 ± 2.94bc	4.89 ± 3.26c	11.21 ± 2.4abc	21.76 ± 8.74a
Geraniol	1.53 ± 0.05bc	0.97 ± 0.06c	1.46 ± 0.38bc	2.93 ± 0.56bc	4.18 ± 0.59b	10.32 ± 2.38a
Geranylacetone	5.12 ± 0.33b	2.29 ± 1.7b	17.39 ± 7.18a	17.66 ± 3.36a	8.87 ± 4.96ab	10.13 ± 0.35ab
Total	91.11 ± 2.81abc	24.99 ± 5.83c	37.59 ± 19.56bc	76.78 ± 31.27bc	97.27 ± 21.98ab	162.83 ± 47.65a
*Norisoprenoids*						
Theaspirane (Isomer 1)	0.32 ± 0.02ab	0.21 ± 0.02b	0.58 ± 0.29a	0.2 ± 0.01b	0.17 ± 0.07b	0.07 ± 0.02b
Theaspirane (Isomer 2)	0.22 ± 0.02b	0.16 ± 0.04b	0.52 ± 0.25a	0.21 ± 0.02b	0.14 ± 0.05b	0.06 ± 0.01b
(*E*)-β-Damascenone	0.32 ± 0.01d	0.43 ± 0.02d	0.77 ± 0.29cd	1.53 ± 0.37bc	2.88 ± 0.65a	2.46 ± 0.26ab
Total	0.86 ± 0.03b	0.81 ± 0.04b	1.87 ± 0.83ab	1.93 ± 0.38ab	3.18 ± 0.76a	2.6 ± 0.28a
*Sesquiterpenoids*						
7-epi-α-Cedrene	2.72 ± 0.73a	1.6 ± 0.34b	0.71 ± 0.06b	0.89 ± 0.12b	1 ± 0.01b	1.15 ± 0.02b
Selina-4,11-diene	0.03±0a	0.01 ± 0b	ND	ND	ND	ND
α-Muurolene	0.07 ± 0.01b	0.11 ± 0.01a	0.05 ± 0.02bc	ND	0.04 ± 0.01bc	0.03±0c
δ-Cadinene	ND	ND	ND	0.11 ± 0.06ab	0.07 ± 0.01b	0.21 ± 0.04a
Calamenene (cis + trans)	0.18 ± 0.02a	0.08 ± 0.01b	ND	0.06 ± 0.01b	0.06 ± 0.01b	0.07 ± 0.01b
α-Calacorene	0.06 ± 0.01a	0.02±0b	ND	0.06 ± 0.02a	0.04±0ab	0.06 ± 0.01a
ω-Cadinene	0.04 ± 0.01a	ND	ND	0.05 ± 0.01a	0.04 ± 0.01a	0.05 ± 0a
γ-Calacorene	0.46 ± 0.03a	0.18 ± 0.01bc	0.06 ± 0.01bc	0.2 ± 0.1b	0.13 ± 0.01c	0.22 ± 0.01b
Palustrol	0.09 ± 0.01a	0.01 ± 0c	ND	0.04 ± 0.01b	0.03 ± 0bc	0.02 ± 0.01bc
1-epi-Cubenol	0.06 ± 0.01b	0.02±0c	0.02±0c	0.13 ± 0.02a	0.09±0b	0.09 ± 0.02b
γ-Eudesmol	0.06 ± 0.01a	0.02 ± 0b	ND	ND	ND	ND
Cubenol	0.05 ± 0.01bc	0.03 ± 0c	0.02 ± 0.01c	0.09 ± 0.03a	0.06 ± 0ab	0.08 ± 0.01ab
Cadalene	0.1 ± 0.02a	0.08 ± 0.01ab	0.02 ± 0c	0.04 ± 0.02c	0.05 ± 0.01bc	0.06 ± 0.02bc
Total	3.94 ± 0.69a	2.15 ± 0.34b	0.89 ± 0.1c	1.67 ± 0.34bc	1.61 ± 0.04bc	2.03 ± 0.04b

Vintage 2017	wpf4	wpf6	wpf8	wpf10	wpf12
*Monterpenoids*					
α-Terpinene	7.38 ± 1.85a	2.27 ± 0.63ab	3.5 ± 0.74b	1.63 ± 0.27ab	0.5 ± 0.25c
Cymene (m- and p-)	2.63 ± 0.84a	0.7 ± 0.08c	2 ± 0.52ab	0.87 ± 0.27bc	0.21 ± 0.07c
1,8-Cineol	1.03 ± 0.59	<0.01	ND	ND	ND
(*E*)-β-Ocimene	0.35 ± 0.08	<0.01	ND	ND	<0.01
γ-Terpinene	15.88 ± 3.79a	5.61 ± 1.04b	11.34 ± 2.05ab	ND	ND
Terpinolene	6.84 ± 1.53a	2.24 ± 0.43bc	3.64 ± 0.81b	1.79 ± 0.32bc	1.03 ± 0.38c
p-Cymenene	0.18 ± 0.01	<0.01	<0.01	<0.01	<0.01
Linalool	3.54 ± 1.1a	1.34 ± 0.1b	1.4 ± 0.15b	1.21 ± 0.23b	4.24 ± 0.78a
Hotrienol	5.49 ± 1.29b	5.88 ± 0.95b	14.82 ± 2.09a	14.3 ± 2.2a	12.51 ± 2.08a
trans-Pinocarveol	ND	<0.01	<0.01	<0.01	<0.01
Citronellal	ND	<0.01	<0.01	<0.01	<0.01
Neroloxide	0.25 ± 0.06c	<0.01	2.46 ± 0.56a	1.55 ± 0.45ab	0.9 ± 0.32bc
Menthol (+isomenthol)	ND	<0.01	<0.01	<0.01	<0.01
Terpinen-4-ol	3.15 ± 0.9a	0.26 ± 0.33b	1.17 ± 0.38b	0.25 ± 0.23b	<0.01
α-Terpineol	6.62 ± 1.29a	2.08 ± 0.49c	4.59 ± 1.1ab	3.33 ± 0.47bc	2.54 ± 0.35bc
Geraniol	1.11 ± 0.02b	0.89 ± 0.18b	0.94 ± 0.04b	1.21 ± 0.28b	3.58 ± 0.39a
Geranylacetone	2.4 ± 0.92b	5.18 ± 0.88a	3 ± 0.39ab	2.2 ± 0.77b	1.9 ± 1.1b
Total	56.58 ± 12.51a	26.61 ± 4.41b	48.86 ± 8.06a	28.35 ± 4.53b	27.44 ± 4.74b
*Norisoprenoids*					
Theaspirane (Isomer 1)	0.16 ± 0.03ab	0.19±0a	0.21 ± 0.04a	0.15 ± 0.02ab	0.11 ± 0.01b
Theaspirane (Isomer 2)	0.17 ± 0.02bc	0.25 ± 0.03a	0.19 ± 0.03ab	0.13 ± 0.03bc	0.11 ± 0.01c
(*E*)-β-Damascenone	0.27 ± 0.03d	0.66 ± 0.12cd	0.99 ± 0.12c	1.74 ± 0.27b	2.74 ± 0.34a
Total	0.59 ± 0.07d	1.1 ± 0.15cd	1.38 ± 0.16c	2.03 ± 0.32b	2.97 ± 0.36a
*Sesquiterpenoids*					
7-epi-α-Cedrene	2.22 ± 0.33ab	1.46 ± 0.19b	2.72 ± 0.92a	1.28 ± 0.13b	1.04 ± 0.06b
Selina-4,11-diene	0.04 ± 0.01a	0.02 ± 0b	ND	ND	ND
α-Muurolene	0.03±0b	0.07 ± 0.01bc	0.13 ± 0.03a	0.08 ± 0.01b	ND
δ-Cadinene	ND	ND	ND	ND	ND
Calamenene (cis + trans)	0.09 ± 0.01a	0.07 ± 0.02a	0.09 ± 0a	0.06 ± 0.01a	0.03±0b
α-Calacorene	0.04 ± 0.01a	0.02 ± 0b	ND	ND	ND
ω-Cadinene	0.05 ± 0a	0.03 ± 0b	0.03 ± 0.01b	ND	ND
γ-Calacorene	0.25 ± 0a	0.24 ± 0.06a	0.21 ± 0.03a	0.2 ± 0.01a	0.11 ± 0.02b
Palustrol	0.12 ± 0.02a	0.02 ± 0.01b	ND	ND	ND
1-epi-Cubenol	0.05 ± 0.01a	0.03 ± 0.01ab	0.03 ± 0.01b	0.02 ± 0b	0.02 ± 0b
γ-Eudesmol	0.02 ± 0a	0.01 ± 0b	0.02 ± 0a	0.01 ± 0b	ND
Cubenol	0.05 ± 0.01a	0.04 ± 0.01a	0.03 ± 0ab	0.02 ± 0.01b	0.01±0b
Cadalene	0.04 ± 0.01b	0.08 ± 0.02a	0.08 ± 0.01a	0.07 ± 0.01a	0.04±0b
Total	2.99 ± 0.36ab	2.1 ± 0.3abc	3.32 ± 0.99a	1.75 ± 0.14bc	1.25 ± 0.07c

**Notes:** Linalool, α-terpineol, geraniol and geranylacetone were quantified using their pure standard compounds. α-terpinene, cymene (m- and p-), 1,8-cineol, (*E*)-β-ocimene, γ-terpinene, terpinolene, p-cymenene and hotrienol were semi-quantified using a linalool standard. All monoterpenes are expressed at μg/g grape sample. All sesquiterpenoids and norisoprenoids were semi-quantified with the internal standard β-cedrene and expressed as equivalent concentrations of the internal standard at μg/kg grape sample. ND: not detected. Values labelled with the same lower case letter in the same row are not significantly (*p* < 0.05) different. Raw data of the table are provide in data file 1.

**Table 8:** Terpene concentrations at different developmental stages of Chardonnay in the two experimental vintages.

Vintage 2016	wpf4	wpf6	wpf8	wpf10
*Monterpenoids*				
α-Terpinene	ND	ND	ND	ND
Cymene (m- and p-)	<0.01	<0.01	<0.01	<0.01
1,8-Cineol	ND	ND	ND	ND
(E)-β-Ocimene	ND	ND	ND	ND
γ-Terpinene	ND	ND	ND	ND
Terpinolene	<0.01	<0.01	<0.01	<0.01
p-Cymenene	<0.01	<0.01	<0.01	<0.01
Linalool	<0.01	0.64 ± 0.6a	<0.01	5.29±5a
Hotrienol	ND	ND	ND	ND
trans-Pinocarveol	<0.01	<0.01	<0.01	7.59 ± 5.79
Citronellal	<0.01	0.63 ± 0.47b	<0.01	13.18 ± 9.92a
Neroloxide	ND	ND	ND	ND
Menthol (+isomenthol)	<0.01	<0.01	<0.01	4.64 ± 3.72
Terpinen-4-ol	ND	ND	ND	ND
α-Terpineol	0.08 ± 0.05a	0.2 ± 0.24a	<0.01	2.4 ± 2.83a
Geraniol	0.88 ± 0.09a	0.83 ± 0.07a	0.92 ± 0.16a	2.22 ± 1.46a
Geranylacetone	5.33 ± 1.42a	3.02 ± 0.13a	13.69 ± 0.82a	18.12 ± 12.05a
Total	6.3 ± 1.46a	4.92 ± 1.36a	14.62 ± 0.98a	53.56 ± 40.31a
*Norisoprenoids*				
Theaspirane (Isomer 1)	0.05 ± 0.01a	0.08 ± 0.04a	0.03 ± 0.01a	ND
Theaspirane (Isomer 2)	0.05 ± 0.01a	0.05 ± 0.02a	0.03 ± 0.01a	ND
(E)-β-Damascenone	0.2 ± 0.04b	0.3 ± 0.03b	0.39 ± 0.04b	2.02 ± 1.04a
Total	0.3 ± 0.05b	0.43 ± 0.08b	0.45 ± 0.04b	2.02 ± 1.04a
*Sesquiterpenoids*				
7-epi-α-Cedrene	1.62 ± 0.44a	1.2 ± 0.58a	0.92 ± 0.2a	1.01 ± 0.02a
Selina-4,11-diene	0.05 ± 0.02a	ND	0.03±0a	ND
α-Muurolene	ND	ND	ND	ND
δ-Cadinene	ND	ND	ND	0.13 ± 0.05
Calamenene (cis + trans)	0.04 ± 0.02b	0.08 ± 0.01a	0.06 ± 0.01ab	0.07 ± 0.01a
α-Calacorene	0.03±0ab	0.02±0b	ND	0.05 ± 0.02a
ω-Cadinene	0.09 ± 0.02a	0.05 ± 0.01b	0.04 ± 0.01b	0.05 ± 0.01b
γ-Calacorene	0.31 ± 0.02a	0.18 ± 0.05a	0.17 ± 0.05a	0.24 ± 0.1a
Palustrol	0.01 ± 0.01b	ND	ND	0.04 ± 0.01a
1-epi-Cubenol	0.06±0b	0.04 ± 0.01b	0.03±0b	0.1 ± 0.02a
γ-Eudesmol	0.04 ± 0.01a	0.03 ± 0.01a	0.03 ± 0.01a	ND
Cubenol	0.02 ± 0b	0.02 ± 0b	0.02 ± 0b	0.07 ± 0.02a
Cadalene	0.09 ± 0.01a	0.1 ± 0.03a	0.07 ± 0.01a	0.06 ± 0.03a
Total	2.36 ± 0.47a	1.71 ± 0.61a	1.34 ± 0.27a	1.83 ± 0.23a

Vintage 2017	4	9	14	19	24
	wpf4	wpf6	wpf8	wpf10	wpf12
*Monterpenoids*					
α-Terpinene	ND	ND	ND	ND	ND
Cymene (m- and p-)	<0.01	<0.01	<0.01	<0.01	<0.01
1,8-Cineol	ND	ND	ND	ND	ND
(E)-β-Ocimene	ND	ND	ND	ND	ND
γ-Terpinene	ND	ND	ND	ND	ND
Terpinolene	<0.01	<0.01	<0.01	<0.01	<0.01
p-Cymenene	<0.01	<0.01	<0.01	<0.01	<0.01
Linalool	<0.01	<0.01	0.09 ± 0.03a	0.2 ± 0.22a	0.37 ± 0.02a
Hotrienol	ND	ND	ND	ND	ND
trans-Pinocarveol	<0.01	<0.01	<0.01	<0.01	<0.01
Citronellal	<0.01	<0.01	0.13 ± 0.08	<0.01	<0.01
Neroloxide	ND	ND	ND	ND	ND
Menthol (+isomenthol)	<0.01	<0.01	<0.01	<0.01	<0.01
Terpinen-4-ol	ND	ND	ND	ND	ND
α-Terpineol	<0.01	<0.01	<0.01	0.34 ± 0.29	<0.01
Geraniol	0.64 ± 0.04b	0.63 ± 0.07b	0.65 ± 0.01b	0.79 ± 0.09b	0.98 ± 0.05a
Geranylacetone	4.5 ± 2.16a	3.83 ± 2.8a	4.77 ± 0.68a	1.08 ± 3.29a	0.64 ± 1.67a
Total	5.14 ± 2.15a	4.47 ± 2.77a	5.61 ± 0.59a	2.23 ± 3.48a	2.01 ± 1.68a
*Norisoprenoids*					
Theaspirane (Isomer 1)	0.05 ± 0.01a	0.04 ± 0.01a	0.03 ± 0ab	0.03 ± 0b	ND
Theaspirane (Isomer 2)	0.05 ± 0.01a	0.04 ± 0.01a	ND	ND	ND
(E)-β-Damascenone	0.19 ± 0.01d	0.33 ± 0.03cd	0.77 ± 0.05c	1.41 ± 0.11b	2.03 ± 0.4a
Total	0.28 ± 0.02d	0.41 ± 0.05cd	0.8 ± 0.05c	1.43 ± 0.11b	2.03 ± 0.4a
*Sesquiterpenoids*					
7-epi-α-Cedrene	2.15 ± 0.4b	1.37 ± 0.17bc	3.31 ± 0.58a	1.17 ± 0.26c	1.15 ± 0.1c
Selina-4,11-diene	0.07 ± 0.02a	0.04±0b	0.03 ± 0.01b	0.02 ± 0.01b	ND
α-Muurolene	ND	ND	ND	ND	ND
δ-Cadinene	ND	ND	ND	ND	ND
Calamenene (cis + trans)	0.12 ± 0.02a	0.04 ± 0.01c	0.08 ± 0.01b	0.06 ± 0.01bc	0.03 ± 0.01c
α-Calacorene	0.03 ± 0.01	ND	ND	ND	ND
ω-Cadinene	0.1±0a	0.04±0b	0.04 ± 0.01b	ND	ND
γ-Calacorene	0.47 ± 0.05a	0.22 ± 0.02bc	0.22 ± 0.02b	0.16 ± 0.07bc	0.12 ± 0.01c
Palustrol	0.05 ± 0.01	ND	ND	ND	ND
1-epi-Cubenol	0.08 ± 0.01a	0.03 ± 0.01b	0.02 ± 0c	0.02 ± 0c	0.01 ± 0c
γ-Eudesmol	0.04 ± 0.01a	0.02 ± 0b	0.03 ± 0.01bc	0.03 ± 0.01bc	0.02 ± 0.01b
Cubenol	0.04 ± 0.01a	0.01 ± 0b	ND	ND	ND
Cadalene	0.09 ± 0a	0.06 ± 0.01abc	0.08 ± 0.01ab	0.05 ± 0.02c	0.04 ± 0c
Total	3.24 ± 0.45a	1.85 ± 0.2b	3.81 ± 0.59a	1.51 ± 0.34b	1.38 ± 0.1b

**Notes:** Linalool, α-terpineol, geraniol and geranylacetone were quantified using their pure standard compounds. α-terpinene, cymene (m- and p-), 1,8-cineol, (*E*)-β-ocimene, γ-terpinene, terpinolene, p-cymenene and hotrienol were semi-quantified using a linalool standard. All monoterpenes are expressed at μg/g grape sample. All sesquiterpenoids and norisoprenoids were semi-quantified with the internal standard β-cedrene and expressed as equivalent concentrations of the internal standard at μg/kg grape sample. ND: not detected. Values labelled with the same lower case letter in the same row are not significantly (*p* < 0.05) different. Raw data of the table are provide in data file 1.

**Table 9:** Terpene concentrations at different developmental stages of Pinot Gris in the two experimental vintages.

Vintage 2016	45	50	55	60
	wpf4	wpf6	wpf8	wpf10
*Monterpenoids*				
α-Terpinene	0.45 ± 0.19a	0.28 ± 0.19a	0.13 ± 0.12a	<0.01
Cymene (m- and p-)	<0.01	<0.01	0.15 ± 0.02a	0.39 ± 0.48a
1,8-Cineol	ND	ND	ND	ND
(*E*)-β-Ocimene	<0.01	ND	ND	ND
γ-Terpinene	2.6 ± 0.68	ND	ND	ND
Terpinolene	0.29 ± 0.2a	0.22 ± 0.11a	0.06 ± 0.07a	<0.01
p-Cymenene	<0.01	<0.01	<0.01	<0.01
Linalool	0.2 ± 0.14b	0.23 ± 0.17b	0.5 ± 0.28b	3.96 ± 1.69a
Hotrienol	<0.01	<0.01	<0.01	<0.01
trans-Pinocarveol	<0.01	<0.01	<0.01	9.27 ± 1.11
Citronellal	<0.01	0.45 ± 0.34b	0.7 ± 0.45b	16.45 ± 2.36a
Neroloxide	<0.01	ND	ND	ND
Menthol (+isomenthol)	<0.01	<0.01	<0.01	5.42 ± 0.91
Terpinen-4-ol	<0.01	<0.01	<0.01	<0.01
α-Terpineol	1.16 ± 0.44a	1.36 ± 0.16a	1.11 ± 0.77a	2.14 ± 0.23a
Geraniol	0.98 ± 0.22b	0.85 ± 0.11b	0.95 ± 0.14b	3.2 ± 1.27a
Geranylacetone	7.41 ± 1.67bc	3.3 ± 1.26c	14.81 ± 3.83ab	18.99 ± 4.58a
Total	13.09 ± 2.05b	6.74 ± 0.74b	18.05 ± 4.72b	59.9 ± 12.76a
*Norisoprenoids*				
Theaspirane (Isomer 1)	0.06 ± 0.01a	0.06 ± 0.02ab	0.03 ± 0.01b	ND
Theaspirane (Isomer 2)	0.07 ± 0.01a	0.04 ± 0.02ab	0.03 ± 0.01b	ND
(*E*)-β-Damascenone	0.19 ± 0.03b	0.31 ± 0.04b	0.56 ± 0.1b	2.69 ± 0.9a
Total	0.33 ± 0.02b	0.41 ± 0.07b	0.62 ± 0.12b	2.69 ± 0.9a
*Sesquiterpenoids*				
7-epi-α-Cedrene	2.04 ± 0.69a	1.61 ± 0.57ab	0.82 ± 0.15b	0.97 ± 0.06ab
Selina-4,11-diene	0.07 ± 0.01a	0.03 ± 0.01b	0.02 ± 0.01b	ND
α-Muurolene	0.09 ± 0.01b	0.17 ± 0.05a	0.08 ± 0.02b	0.04 ± 0.02b
δ-Cadinene	ND	ND	ND	0.12 ± 0.03
Calamenene (cis + trans)	0.11 ± 0.01a	0.11 ± 0a	0.08±0b	0.08±0b
α-Calacorene	0.05 ± 0a	0.03 ± 0a	ND	0.06 ± 0.02a
ω-Cadinene	0.07 ± 0a	0.03 ± 0c	ND	0.05 ± 0.01b
γ-Calacorene	0.31 ± 0a	0.3 ± 0.09a	0.14 ± 0.05a	0.2 ± 0.08a
Palustrol	0.04 ± 0.01a	ND	ND	0.04±0a
1-epi-Cubenol	0.06 ± 0.01b	0.03 ± 0.01c	0.02 ± 0.01c	0.13 ± 0.02a
γ-Eudesmol	0.04 ± 0.01a	0.04±0a	0.02 ± 0.01a	ND
Cubenol	0.05±0b	0.04 ± 0.01bc	0.02 ± 0.01c	0.09 ± 0.01a
Cadalene	0.08 ± 0.01ab	0.11 ± 0.02a	0.06±0b	0.06 ± 0.03b
Total	3.01 ± 0.71a	2.5 ± 0.68ab	1.26 ± 0.21b	1.84 ± 0.22ab

Vintage 2017	5	10	15	20	25
	wpf4	wpf6	wpf8	wpf10	wpf12
*Monterpenoids*					
α-Terpinene	3.5 ± 0.55a	0.99 ± 0.29b	<0.01	<0.01	0.06 ± 0.03c
Cymene (m- and p-)	0.74 ± 0.21	<0.01	<0.01	<0.01	<0.01
1,8-Cineol	ND	ND	ND	ND	ND
(*E*)-β-Ocimene	<0.01	ND	ND	ND	ND
γ-Terpinene	8.98 ± 1.01a	3.95 ± 0.86b	ND	ND	ND
Terpinolene	2.29 ± 0.47a	0.53 ± 0.23b	<0.01	<0.01	0.07 ± 0.04c
p-Cymenene	<0.01	<0.01	<0.01	<0.01	<0.01
Linalool	1.1 ± 0.25a	0.41 ± 0.12b	0.05 ± 0.02b	1.54 ± 0.32a	1.37 ± 0.05a
Hotrienol	0.57 ± 0.14a	0.2 ± 0.11b	<0.01	<0.01	0.1 ± 0.14b
trans-Pinocarveol	<0.01	<0.01	<0.01	<0.01	<0.01
Citronellal	<0.01	<0.01	0.16 ± 0.12a	<0.01	0.18 ± 0.05a
Neroloxide	<0.01	<0.01	<0.01	ND	ND
Menthol (+isomenthol)	<0.01	<0.01	<0.01	<0.01	<0.01
Terpinen-4-ol	1.18 ± 0.45	<0.01	<0.01	<0.01	<0.01
α-Terpineol	2.76 ± 0.64a	0.68 ± 0.21b	<0.01	1.31 ± 0.15b	0.86 ± 0.28b
Geraniol	0.82 ± 0.0c4	0.78 ± 0.07c	0.86 ± 0.14c	1.58 ± 0.19b	2.24 ± 0.19a
Geranylacetone	4.46 ± 0.92bc	4.57±1b	7.49 ± 1.4a	2.02 ± 0.49bc	1.64 ± 1.33c
Total	26.43 ± 4.27a	12.22 ± 2.16b	8.51 ± 1.55b	6.66 ± 0.46b	6.49 ± 1.18b
*Norisoprenoids*					
Theaspirane (Isomer 1)	0.07 ± 0.01a	0.05 ± 0.02ab	0.04±0b	0.03 ± 0.01b	ND
Theaspirane (Isomer 2)	0.06 ± 0.01a	0.06 ± 0.01ab	0.04±0bc	0.03±0c	ND
(*E*)-β-Damascenone	0.28 ± 0.02c	0.42 ± 0.03c	0.6 ± 0.04b	1.38 ± 0.06a	1.4 ± 0.11a
Total	0.41 ± 0.04c	0.52 ± 0.03bc	0.68 ± 0.04b	1.43 ± 0.07a	1.4 ± 0.11a
*Sesquiterpenoids*					
7-epi-α-Cedrene	3.16 ± 0.53a	1.78 ± 0.14b	1.16 ± 0.01b	1.7 ± 0.3b	1.17 ± 0.02b
Selina-4,11-diene	0.07 ± 0.01a	0.05 ± 0.02ab	0.02 ± 0.01b	0.03 ± 0.02b	ND
α-Muurolene	0.04 ± 0.01b	0.08 ± 0.02a	0.06 ± 0.01ab	0.09 ± 0.02a	ND
δ-Cadinene	ND	ND	ND	ND	ND
Calamenene (cis + trans)	0.1 ± 0.01a	0.11 ± 0.02a	0.06 ± 0b	0.1 ± 0.01a	0.05 ± 0.01b
α-Calacorene	0.03±0a	0.03±0a	0.02 ± 0b	0.02 ± 0b	ND
ω-Cadinene	0.06 ± 0.01a	0.03 ± 0.01b	0.02±0c	ND	ND
γ-Calacorene	0.25 ± 0.03a	0.27 ± 0.03a	0.19 ± 0.01bc	0.24 ± 0.01ab	0.16 ± 0.02c
Palustrol	0.07 ± 0.01a	0.03±0b	ND	ND	ND
1-epi-Cubenol	0.05 ± 0.01a	0.03 ± 0.01ab	0.02 ± 0b	0.02 ± 0b	0.02 ± 0.01b
γ-Eudesmol	0.03 ± 0.01a	0.02 ± 0ab	0.01±0b	0.03 ± 0a	ND
Cubenol	0.04±0a	0.04 ± 0.02a	0.02±0a	0.03 ± 0a	ND
Cadalene	0.03 ± 0.01d	0.09 ± 0.01a	0.07 ± 0.01bc	0.09 ± 0.01ab	0.05 ± 0.01cd
Total	3.93 ± 0.47a	2.57 ± 0.1b	1.65 ± 0.01cd	2.36 ± 0.35bc	1.45 ± 0.04d

**Notes:** Linalool, α-terpineol, geraniol and geranylacetone were quantified using their pure standard compounds. α-terpinene, cymene (m- and p-), 1,8-cineol, (*E*)-β-ocimene, γ-terpinene, terpinolene, p-cymenene and hotrienol were semi-quantified using a linalool standard. All monoterpenes are expressed at μg/g grape sample. All sesquiterpenoids and norisoprenoids were semi-quantified with the internal standard β-cedrene and expressed as equivalent concentrations of the internal standard at μg/kg grape sample. ND: not detected. Values labelled with the same lower case letter in the same row are not significantly (*p* < 0.05) different. Raw data of the table are provide in data file 1.

## 2 Experimental design, materials and methods

Wine grape samples were harvested from a commercial vineyard in the Grampians wine region in Victoria, Australia. In two experimental vintages, vertical shoot positioned (VSP) trellis and drip irrigation systems were applied in the vineyard without significant pest or disease pressure detected. In vintage 2016, sample collection started from 18 December 2015 and continued in two-week intervals until commercial harvest. Matured Chardonnay and Pinot Gris were collected on 13 February 2016, while Shiraz, Cabernet Sauvignon and Riesling were collected on 14 March 2016. In vintage 2017, samples were collected fortnightly from 09 January 2017 due to delayed fruit-setting. The last batches of Riesling, Chardonnay and Pinot Gris were harvested on 20 March 2017 while Shiraz and Cabernet Sauvignon were on 18 April 2017. For each cultivar, grape brunches in triplicate were collected randomly from different positions of randomly selected grapevines (n > 30 for each cultivar). Samples were transported to the laboratory on dry ice and stored at −20 °C before analysis.

Terpene analysis was conducted on an Agilent 6890 GC coupled with an Agilent 5973 MSD (Agilent Technologies, Santa Clara, CA) and an Agilent PAL multipurpose sampler connected to the GC. The HS-SPME-GC-MS analysis was conducted based on our previous data with some modifications [3].

Briefly, after destemming, grape berries were frozen with liquid nitrogen and then powdered with a stainless steel grinder. Five g of the sample powder was extracted with 30 mL of a pH 3.2 extraction solution, which consisted of 5 g/L polyvinylpolypyrrolidone (PVPP), 0.5 g/L tartaric acid and 0.5 g/L of sodium sulfite, at room temperature for 24 h with a stirring rate of 100 rpm. A 0.45 μm nylon syringe filter was used to filter the mixture and then 5 mL of the supernatant was mixed with 1 g of sodium chloride and 20 μL of 2 mg/L β-cedrene internal standard in a 20 mL GC vial. The headspace in the GC vial was extracted by using a 65 μm DPMS/DVB SPME fibre (Supelco, Bellefonte, PA) for 60 min with agitation at 45 °C in an agitator mounted on the Agilent PAL multipurpose sampler.

Chromatographic separation was achieved on a J&W DΒ-5ms capillary column (Agilent Technologies; 30 m × 0.25 mm × 0.25 μm). Purified helium was used as the carrier gas at a constant flow rate of 1.0 mL/min. GC conditions were based on our previous protocol with slight modifications (Zhang et al., 2016). Compounds adsorbed on the SPME fibre were desorbed under pulsed splitless mode and the mass spectrometer was operated in scan/sim mode under positive electron ionization (EI) mode at 70 eV, with a scan range from *m/z* 35 to 280.

As sesquiterpenes exist in trace amounts, simultaneous selected ion monitoring (SIM) mode was used to record common terpene ions: *m/z* 105, 133, 147, 161, and 204 to facilitate locating target compounds. Quantification of terpenes was based on the target ion peak areas.

A mixed alkane standard (C7–C30) was used to determine the retention index (RI; *I*) for each peak. Terpene identification was carried out by matching the mass spectrum and *I* value in the terpenoids library using MassFinder 4 software (Hochmuth Scientific Consulting, Hamburg, Germany). Although *I* values in the terpenoids library are based on a J&W DB1 column while in this present data a DB-5 column was used, these two nonpolar columns have very close *I* profiles as shown in previous data [4]. Therefore, *I* values from the terpenoids library are reliable in facilitating the tentative identification in the present data. Peak integration was then conducted with the Agilent ChemStation software based on the responses of target ions. Calculated and reference RI are summarized in Table 3. Mass spectra of semi-quantified terpenes are provided in Table 4. Quantification of each terpene was performed against calibration curves constructed by a series of standards including, α-terpineol, linalool, geraniol, geranylacetone. α-Terpinene, cymene (m- and p-), 1,8-cineol, (*E*)-β-ocimene, γ-terpinene, terpinolene, p-cymenene and hotrienol were semi-quantified using a linalool standard. All sesquiterpenoids and norisoprenoids were semi-quantified with the internal standard β-cedrene and expressed as equivalent concentrations.

Significant (*p* < 0.05) differences in terpene concentrations at different development stages of each grape cultivar were analysed by one-way ANOVA using SPSS 24 (SPSS Inc., Chicago, IL).
